# Experimental Investigation of Bubble Migration near Anisotropic Beams

**DOI:** 10.3390/mi12121518

**Published:** 2021-12-06

**Authors:** Zhicheng Xu, Xiaojian Ma, Qidong Yu, Jing Zhao, Dapeng Wang, Xiaosheng Bi, Fen Qin

**Affiliations:** Department of Research and Development, China Academy of Launch Vehicle Technology, Beijing 100076, China; littlecz@163.com (Z.X.); yuqidong1985@163.com (Q.Y.); sand810125@163.com (J.Z.); wangdapeng1990@hrbeu.edu.cn (D.W.); bixiaosheng@hrbeu.edu.cn (X.B.); qinfen@163.com (F.Q.)

**Keywords:** bubble migration, anisotropic beam, bending-twisting coupling effect

## Abstract

In order to resist bubble loading, anisotropic composite materials are the development direction of the future. The objective of this paper was to experimentally investigate the hydrodynamic performance of anisotropic laminate composite plates, with a focus on the effect of its anisotropic characteristics on single bubble migration. In these experiments, the bubble was generated in a transparent water tank filled with sufficiently degassed water by Joule heating at the connecting point of the electrodes through the discharge of a 6600 μF charge to 800 V, and a high-speed camera system with a recording speed of 40,000 frames per second was used to record the temporal evolution of bubble patterns and the dynamic responses of the laminated composite plates. The results are presented for two anisotropic cantilever composite beams with different ply angles, namely, 0° and 30°. Several variables, such as the shapes of the bubble, the curved trail of motion of the bubble center, bubble collapse time, and bubble initial standoff distances were extracted from the photographic images. The results showed that bubble migration near the 30° plate presents a curved bubble trail with an evident tilted angle during the collapse and rebound stages, which is very different from bubbles that all move vertically above the 0° plate. Furthermore, a characterization method for bubble migration was proposed to quantitatively describe the curved bubble trails and the deformation of the composite beams in temporal and spatial scales. This method shows that the curved bubble trails near the 30° plate are closely related to the dynamic response of composite beams, with a focus on the bending-twisting coupling effect.

## 1. Introduction

The occurrence of bubble collapse near the surface of fluid machinery is a devastating phenomenon with several consequences, such as cavitation erosion [1,2], structural vibration [3,4], and noise emission [5,6]. These destructive mechanical phenomena result from the strong and nonlinear load caused by the high-speed jet and bubble migration [7,8]. It is proven that traditional materials, such as metal and alloy, cannot meet the extreme mechanical requirements caused by bubble collapse [9,10]. In recent decades, inspired by an interesting phenomenon in which bubbles migrate to a rigid wall and are repelled away by the free surface [11], engineers attempt to find a flexible material to repel the bubble migration and high-speed jet away to prevent the collapsing bubble load.

In recent years, carbon-fiber-reinforced resin composite materials have played an important role in structural applications due to their high specific strength and stiffness as compared to plastics, alloys, metals, or other common structural materials [12]. Due to its further advantages of bubble load resistance, anisotropy, and designable ability, this kind of composite has been of much interest in fields involving fluid mechanics, such as in ship propellers [13] and pumping machinery [14], in order to prevent the presence of bubble behaviors and improve the efficiency of operations [15].

The study of bubble–composite interactions began with the works of Shima et al. [16] and Tomita et al. [17], where they experimentally investigated the effect of sandwich structure on bubble dynamics, especially for bubble migration. In recent years, Gong et al. [18,19] used both numerical and experimental approaches to deal with the interaction between a spark-generated bubble and a two-layered composite beam (constrained at its two ends), which consists of an aluminum sheet coated by an elastic layer. Here, they found that the bubble collapse time was greatly influenced by the nearby two-layered boundary. Wang et al. [20] applied a numerical method to evaluate the dynamic response of laminated plates subjected to underwater shock. The results suggested that fluid–solid interaction (FSI) effects have a significant influence on the reaction of the laminated plate, and that the responses of air-backed plates are always stronger than water-backed plates. However, the laminated composite plates mentioned above are all isotropic materials. With composite laminates, the orientation of each ply within the laminate contributes to a global structural response based on its orientation. Arbitrarily oriented lamina within a laminate can induce multiple couplings if the laminate is asymmetric and unbalanced about the laminate mid-line. Bend-twist coupling occurs especially when the laminate is ply-symmetric about the mid-line, but unbalanced, due to its anisotropic properties. Hsiao et al. [21] used a numerical method to investigate the interaction between an underwater explosion and a composite propeller. Fiber orientation in the various layers was studied to understand which combinations of materials and fiber orientations give the strongest resistance in terms of both the bending and twisting of the blade. The bending effect of structure on bubble dynamics was reported by Gong et al. [22]; therefore, we focus on the bending-twisting effect of laminated plate on bubbles in this paper.

In the present work, a systematic investigation of the effect of anisotropic laminated composite beams on the dynamics of spark-charged bubbles was carried out experimentally. The detailed shape variations of bubble migration, the bending-twisting coupling of the structure, and bubble oscillation time were analyzed. The objectives of this paper were to (1) demonstrate the transient evolution process of the bubble near the anisotropic beams, especially for bubble migration and (2) analyze the relationship between bubble migration and the dynamic response of composite beams, with a focus on the bending-twisting effect.

## 2. Experimental Setup

### 2.1. Bubble Generator

The Joule heating method was used to generate a single bubble in the liquid. The high voltage pulse was supplied by a 6600 μF capacitor charged to 800 V. Two copper electrodes were arranged in a cross configuration overlapping with each other. Upon discharge, a single vapor bubble was generated at the connecting point of the electrodes due to the gasification. A charge coupled device (CCD) high-speed camera (HG-LE, by Redlake) was used to record the temporal evolution of the bubble dynamics with a recording speed of 40,000 frames per second. Before the formal test, a related repeatability test was performed and the results showed that the bubble profile produced differed by about 5% each time, leading us to believe that this bubble generator has a stable and strong repeatability. A continuous light source located at one side of the water tank provided the light illumination for the camera. To achieve the synchronous trigger, the camera, lamp, and power supply were controlled by a delay generator at the same time. The detailed information about the experimental setup can be referred to in our previous work [12].

In order to quantitatively measure the initial position of bubbles, a normalized initial position between the bubble and the boundary was defined as:

(1) $$\gamma =\frac{L}{R_{m}}$$

where *L* is the distance from the bubble center to the boundary at inception and *R_m_* is the maximum radius of the bubble [12].

### 2.2. Anisotropic Beams

**Figure 1:**
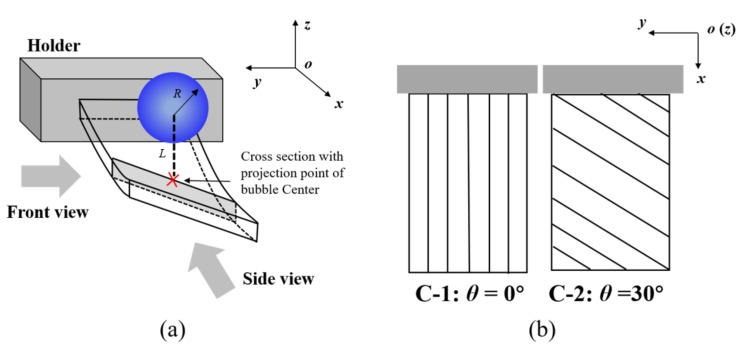
Schematic diagram of (**a**) fixation mode and (**b**) fiber ply angle of anisotropic composite beams.

In order to study the coupling effect of bending and twisting at the ends of anisotropic composite materials, Figure 1a shows the fixation mode and fiber ply angle of the anisotropic composite plate. The thickness of the composite board was 1.5 mm with 8 carbon-fiber layers. The board was machined to *a* = 120 mm in length and *b* = 75 mm in width with the shorter end of the plates constrained by a holder. The short side of the wall was set as a free end, and only one side of the wall was fixed. The whole structure wall can be regarded as cantilever beam. The high-speed camera was regarded as the front view along the negative direction of the *y*-axis, and the side view along the negative side of the *x*-axis. It should be noted that the projection position of the initial position of the bubble on the wall was the center of the short side and was 25 mm away from the free end.

In addition, the fiber ply angle of the C-1 beam was *θ* = 0° and that of the C-2 beam was *θ* = 30°. The relative relationship between the composite fiber ply angle and the whole coordinate system is shown in Figure 1b, and its detailed mechanical properties can be reviewed in Table 1. Therefore, according to the macroscopic mechanical analysis, the C-1 plate only has a bending effect, while C-2 has a bending-twisting coupling effect. By comparing the bubble morphology and migration direction near the wall of the C-1 and C-2 anisotropic composites, the effect of bending-twisting coupling on bubble migration can be explored.

## 3. Results and Discussion

### 3.1. Temporal Evolution of Global Bubble Shapes

**Figure 2:**
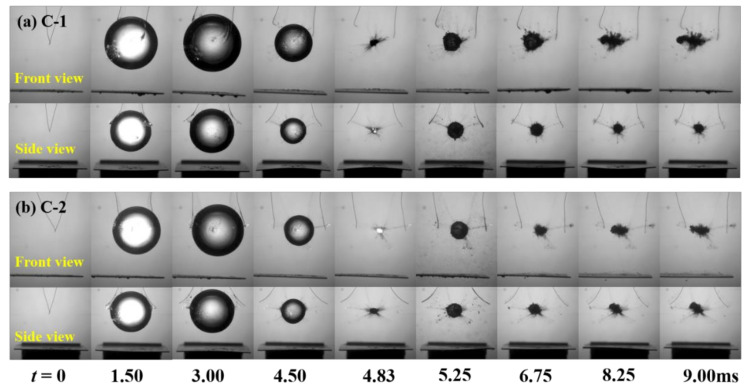
Front view and side view of the transient evolution of the bubble near (**a**) C-1 and (**b**) C-2 plates (*γ* = 1.65).

**Figure 3:**
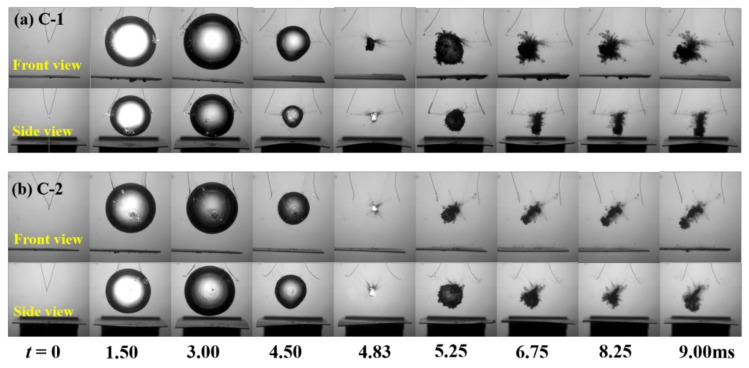
Front view and side view of the transient evolution of the bubble near (**a**) C-1 and (**b**) C-2 plates (*γ* = 1.32).

**Figure 4:**
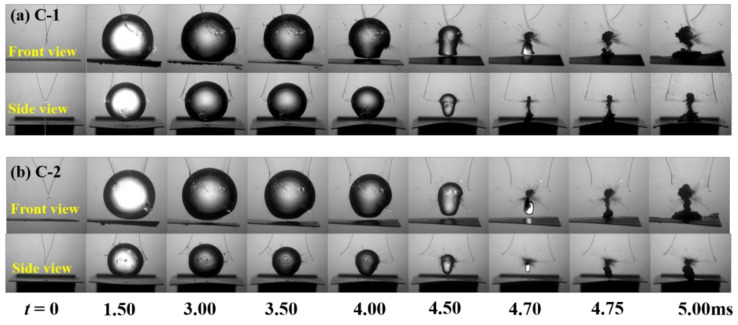
Front view and side view of the transient evolution of the bubble near (**a**) C-1 and (**b**) C-2 plates (*γ* = 1.00).

In order to show the effect of the anisotropic beams on bubble shapes, Figure 2, Figure 3 and Figure 4 show the transient evolution process of bubble morphology at three different initial bubble positions, i.e., *γ* = 1.65, 1.32, and 1.00. When the normalized parameter *γ* is more than 1.65, the cases show almost no specific phenomenon, but instead show the buoyancy effect.

Figure 2 shows the front and side views of the transient evolution of the shape of the bubble near the C-1 and C-2 plates when *γ* = 1.65. As shown in Figure 2a, it can be observed from the front view that when the time was *t* = 0–4.83 ms (the first bubble oscillation period), the bubble near the C-1 plate expanded and contracted as a sphere. When the time was *t* = 3.00 ms, the bubble reached its maximum volume, and when the time was *t* = 4.83 ms, the bubble shrunk to its minimum volume. It should be noted that during the first oscillation period, the center of the bubble moved obviously. When the time was *t* = 5.25–10.50 ms, the bubble expanded and contracted many times. In this process, the center position of the bubble began to change and moved to the left (negative *x*-axis direction). From the side view, it can be seen that the bubble expanded and contracted as a sphere during the whole observation period, and there was no obvious migration movement. In contrast, when the bubble oscillated near the C-2 plate, the front view showed that the bubble expanded, contracted, and collapsed in the first cycle with time *t* = 0–4.83 ms. In this process, the bubble did not move obviously. When *t* = 6.70–10.50 ms, the migration trajectory of the bubble was slightly upward to the left. The side view shows that when the time was *t =* 0–4.83 ms, the bubble still expanded, contracted, and collapsed as a sphere in the first cycle. When *t* = 6.70–10.50 ms, the bubble moved to the left and slightly upward.

Figure 3 shows the front and side views of the transient evolution of the bubble near the C-1 and C-2 plates when *γ* = 1.32. Similarly, in the period of *t* = 0–3.00 ms, the bubble oscillated from the C-1 plate and C-2 plate with a spherical expansion. The bubble kept in situ oscillation during *t* = 4.50–4.83 ms, and its center position did not change. When the time was *t* = 5.25–9.00 ms, the second cycle of expansion and collapse of the bubble began near the C-1 and C-2 plates. The difference was that, at the beginning of the second collapse cycle, the bubble near the C-1 plate migrated to the left and down in the front view, but moved directly downward in the side view. The bubble near the C-2 plate moved to the left and down in the front view, and also moved to the left and down in the side view.

Figure 4 shows the front and side views of the transient evolution of the bubble near the C-1 and C-2 plates when *γ* = 1.00. In the front view of the C-1 plate, the bubble reached its maximum volume at *t* = 1.50 ms, the bottom surface of the bubble contacted the wall structure, and the free end of the C-1 plate obviously moved downward. When the time was *t* = 3.00–4.50 ms, due to the oscillation of the plate, the bubble gradually formed a mushroom shape. However, due to the larger displacement of the free end than that of the fixed end, the deformation of the right side of the bubble was larger than that of the left side. When the time was *t* = 4.70–5.00 ms, the second cycle of the expansion process started from the lowest volume. In this process, the bubble split into two parts and the upper part of the sub bubble moved upward to the left, while the lower molecular bubble adhered to the surface of the structure wall, and the left side of the lower molecular bubble was higher than the right side. For the front view of the C-2 panel, although the fiber ply angle of the wall structure is different, the bubble collapse morphology was very similar to the bubble near the C-1 plate, so we will not repeat here. In the side view of C-1 plate, the bubble expansion, collapse, and rebound stages were very similar to those near the elastic boundary, resulting in a symmetrical mushroom shape. In addition, the mushroom-like bubble formed two sub-bubbles with a vertical wall structure and motion in opposite directions. In the side view of the C-2 plate, an asymmetric mushroom-like deformation on the right side was larger than that on the left side in the first oscillation period (*t* = 0–4.5 ms). When the time was *t* = 4.70–5.00 ms, the bubble split into two sub-vacuoles. The lower sub-bubble was attached to the surface of the structure wall, the upper terminal bubble was a point-like bubble, and migration occurred.

**Figure 5:**
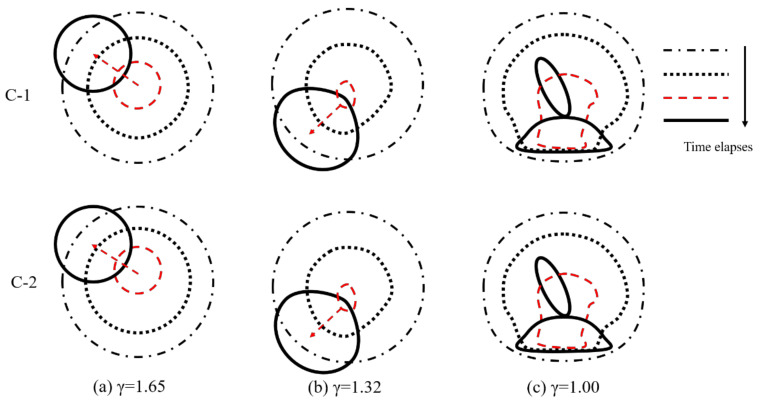
Comparison of bubble collapse patterns in front view near C-1 and C-2 plates under different initial distances (**a**) *γ*=1.65, (**b**) *γ*=1.32, (**c**) *γ*=1.00.

**Figure 6:**
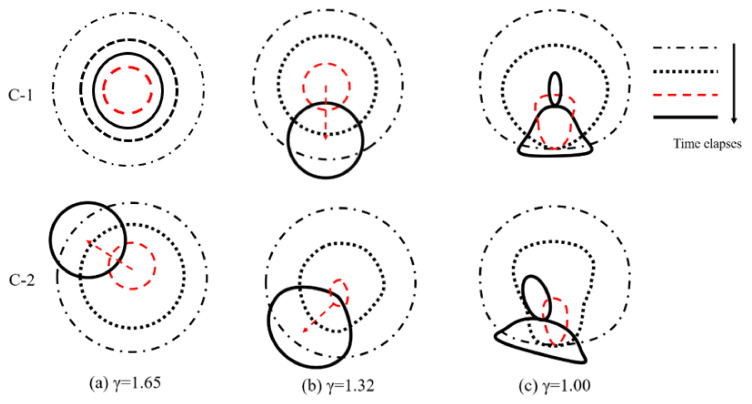
Comparison of bubble collapse patterns in side view near C-1 and C-2 plates under different initial distances (**a**) *γ*=1.65, (**b**) *γ*=1.32, (**c**) *γ*=1.00.

In order to clearly and intuitively describe the bubble morphology, the comparison of the front and side views of bubble collapse near the C-1 plate and the C-2 plate is given in Figure 5 and Figure 6, respectively. For the front view shown in Figure 5, the bubble morphology and migration direction near the C-1 and C-2 plates were similar to each other. When *γ* = 1.65, the bubble moved to the upper left; when *γ* = 1.32, the bubble moved to the lower left; and when *γ* = 1.00, the bubble split into two parts, and the upper molecular bubble moved to the upper left while the lower molecular bubble adhered to the surface of the structure wall. For the side view shown in Figure 6, all the bubbles near the C-1 plate migrated along the direction of gravity, but the direction of bubble migration near the C-2 plate was deflected. The experimental results show that the bending effect of the C-1 plate makes the trajectory of the bubble deflect and migrate only in the front view, which makes a certain angle with the direction of gravity. In contrast, due to the bending-twisting coupling effect of the C-2 plate, the migration trajectory of the bubble makes an angle with the direction of gravity in the front view and the side view.

**Figure 7:**
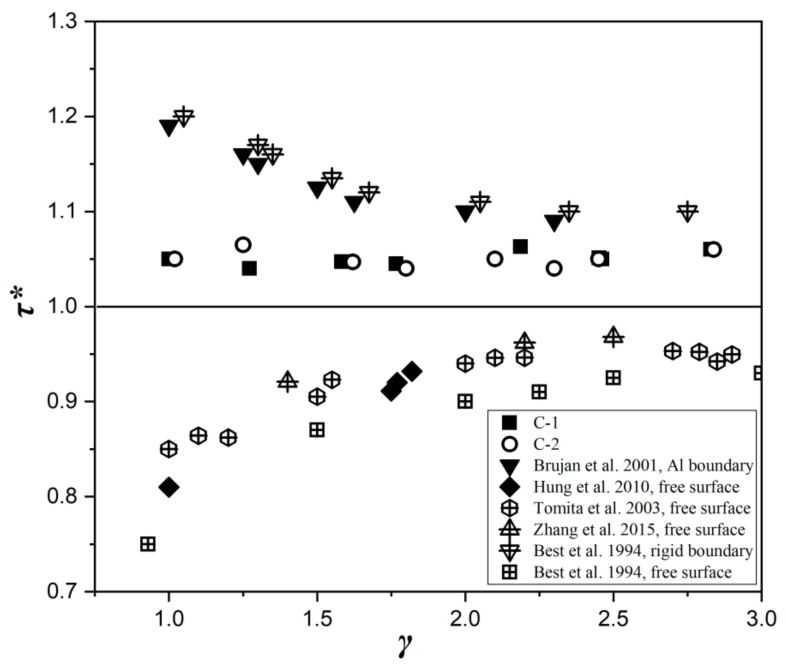
Comparison of bubble collapse time near composite plates with different ply angles.

Figure 7 shows the comparison of bubble collapse times near the C-1 and C-2 plates. The normalized time of bubble collapse *τ∗* is defined as:

(2) $$\tau^{*}=\frac{t_{B}}{t_{OSC}}$$

where *t_B_* is the time from bubble inception to minimum volume, and *t_OSC_* is defined as the Rayleigh oscillation time of the bubble. In order to compare this value with other typical wall collapse times, the bubble collapse time near a rigid wall reported by Brujan et al. [23] and Best et al. [24], and the bubble collapse time near a free surface reported by Hung and Hwangfu [15], Tomita et al. [17], Zhang et al. [25], and Best et al. [24] are also included in the figure. As shown in the figure, the bubble collapse time near the C-1 and C-2 plates is between the values for collapse near the rigid wall and near the free surface. At the same time, the collapse time of the bubble near the C-1 plate and the C-2 plate is slightly longer than one for different initial positions of the bubble. However, the collapse time of the bubble near C-1 and C-2 was consistent, which indicates that the coupling effect of bending and twisting of the C-1 plate does not affect the collapse time of the bubble.

### 3.2. Characterization Method of Curved Bubble Migration

**Figure 8:**
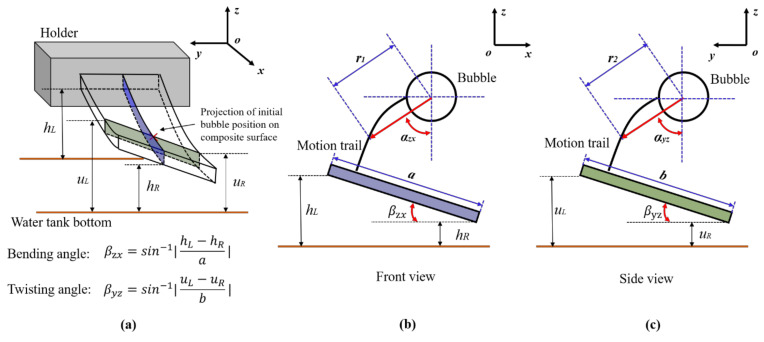
Schematic diagram of bubble trajectory and composite beam response: (**a**) 3D schematic diagram of bending and twisting angle definition; (**b**) bending angle of composite beam and bubble trajectory parameters, (**c**) twisting angle of composite beam and bubble trajectory parameters.

In order to quantitatively describe the corresponding relationship between the bubble curve migration trajectory and the bending-twisting coupling effects of composite materials, the bending angle, twisting angle, and bubble trajectory parameters of the composite plate are shown in Figure 8. The response process of the board was measured by an image-processing method. As shown in Figure 8b, the bending angle of composite plate is defined as:

(3) $$\beta_{xz}=\sin^{-1}\left|\frac{h_{L}-h_{R}}{a}\right|$$

where *h_L_* and *h_R_* are the heights of the two ends of the long side of the composite plate from the bottom of the water tank. The trajectories of the bubble are mapped to a curve in the front view:

(4) $$\left\{\begin{array}{l} \left(x,z\right)=\left(r_{1}\cos\alpha_{zx},r_{1}\sin\alpha_{zx}\right)\\ r\sim r(t)\\ \alpha_{zx}\sim \alpha_{zx}(t) \end{array}\right.$$

where *r*_1_ is the linear distance mapped by the trajectory of bubble migration in the front view, and *α_xz_* is the angle between the line *r*_1_ and the direction of gravity. It is worth noting that both *r*_1_ and *α_xz_* are time dependent variables. Similarly, as shown in Figure 8c, the twisting angle of composite plate is defined as:

(5) $$\beta_{yz}=\sin^{-1}\left|\frac{u_{L}-u_{R}}{b}\right|$$

where *u_L_* and *u_R_* are the height from the two ends of the short side of the composite plate to the bottom of the water tank. The mapping of the bubble migration trajectory in the side view can be defined as:

(6) $$\left\{\begin{array}{l} \left(y,z\right)=\left(r_{2}\cos\beta_{yz},r_{2}\sin\beta_{yz}\right)\\ r\sim r(t)\\ \beta_{yz}\sim \beta_{yz}(t) \end{array}\right.$$

where *r*_2_ is the linear distance mapped by the trajectory of bubble migration in the side view, and *α_yz_* is the angle between the line *r*_2_ and the direction of gravity.

**Figure 9:**
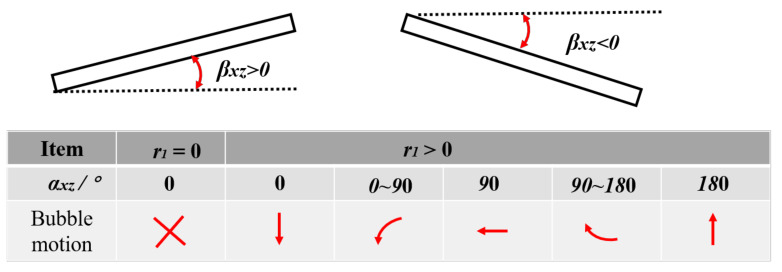
Physical meanings of bending angle (*β_xz_*) of composites and bubble migration trajectory (r_1_, *α_xz_*).

**Figure 10:**
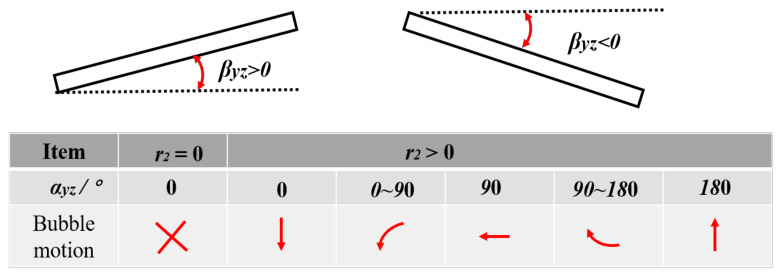
Physical meanings of bending angle (*β_yz_*) of composites and bubble migration trajectory (r_2_, *α_yz_*).

In order to clearly understand the physical meaning of the variables *β_xz_*, *r*_1_, α, and *α_zx_*, the physical meaning of the mapping trajectory (*r*_1_, *α_zx_*) between the bending angle *β_xz_* and the bubble migration in the front view is given in Figure 9. For *β_xz_*, when *β_xz_* > 0, the position of the left endpoint of the middle section of the long side of the composite material is higher than that of the right endpoint, indicating that the free end of the composite plate has risen; when *β_xz_* < 0, the position of the right endpoint of the middle section of the long side of the composite material is higher than the left endpoint, indicating that the free end of the composite plate has sunk. Since *r*_1_ and *α_xz_* jointly determine the position of the bubble migration at a certain time, the physical relationship between them must be considered at the same time. The values of *r*_1_ = 0 and *α_zx_* = 0 indicate that the bubble oscillates at a fixed position and there is no migration. Under the condition of *r*_1_ > 0, when *α_xz_* is 0°, 90°, and 180° the bubble migrates downward, leftward, and upward, respectively; when 0 < *α_xz_* <90°, the bubble migrates to the left and down; when 90°< *α*_xz_ < 180°, the bubble migrates to the upper left. Similarly, Figure 10 gives the physical meaning of the mapping trajectory (*r*_2_, *α_yz_*) between the twisting angle (*β_yz_*) of the composite and the bubble migration trajectory (*r*_2_, *α_yz_*) in the side view.

### 3.3. Effect of Bending-Twisting Coupling of Plate on Bubble Migration

**Figure 11:**
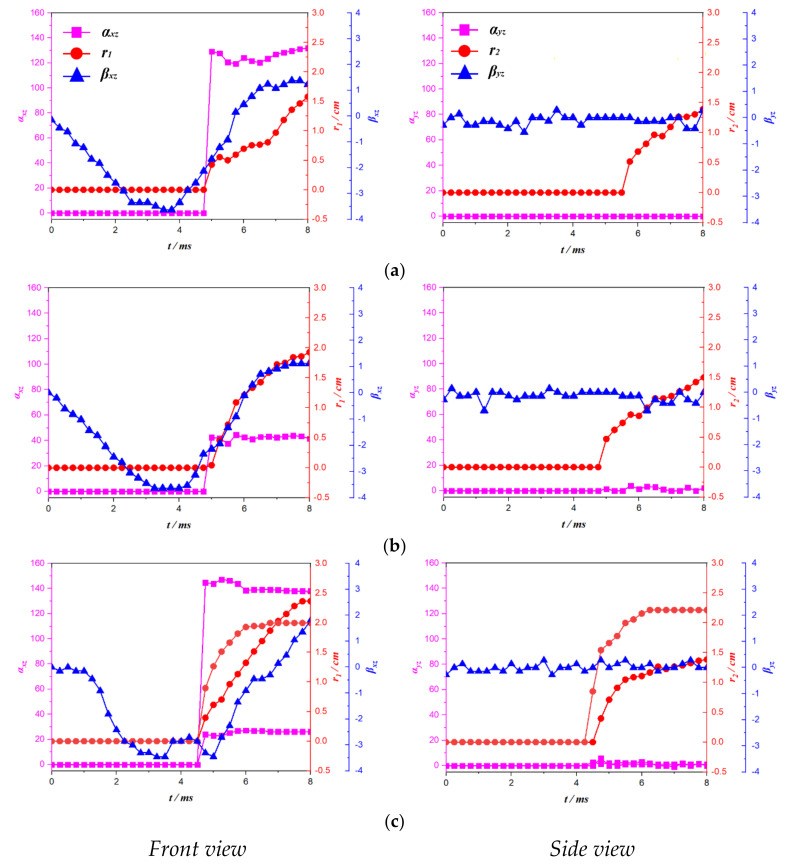
Effect of bending of C-1 plate at different initial positions on the direction of bubble migration. (**a**) *γ* = 1.65, (**b**) *γ* = 1.32, (**c**) *γ* = 1.00.

Figure 11 shows the relationship between the bending effect of the C-1 plate at different initial positions of the bubble and the direction of bubble migration. When the initial position of the bubble is *γ* = 1.65, the trajectory of the bubble and the deformation of the C-1 plate are observed and projected on the front view. When the time is *t* = 0–4.83 ms, the length and deflection angle of the bubble trajectory are *r*_1_ = 0 and *α_zx_* = 0, respectively, which indicates that the bubble oscillates in situ during this period; meanwhile, the bending angle *β_zx_* < 0° of the C-1 plate indicates that the free end of C-1 plate is in the process of subsidence, and the value decreases first and then increases. When the time is *t* = 4.83–8.00 ms, values of *r*_1_ > 0 and 90° < *α_zx_* < 180° indicate that the bubble moves to the left and up; the bending angle (*β_zx_*) of the C-1 plate is less than 0° at first, and then greater than 0° after *t* = 6.0 ms, indicating that the free end of the C-1 plate changes from a sinking state to an upward state. The trajectory of the bubble and the deformation of the C-1 plate were observed quantitatively in the lateral view. It was found that the twisting angle of the C-1 plate always fluctuated up and down at *β_yz_* = 0° during the whole process of motion. This shows that there was no twisting effect of the C-1 plate under the action of bubble collapse. When the time is *t* = 0–5.5 ms, the length and deflection angle of the bubble trajectory are *r*_2_ = 0 and *α_yz_* = 0°, respectively, indicating that there is no migration of the bubble in this time period; when the time is *t* = 5.5–8.0 ms, the *r*_2_ > 0 and *α_yz_* = 0° values of the bubble indicate that the direction of the bubble migration is vertically downwards.

When the initial position of the bubble is *γ* = 1.32, the trajectory of the bubble and the deformation of the C-1 plate are observed and projected on the front view. When the time is *t* = 0–4.83 ms, the length and deflection angle of the bubble trajectory are *r*_1_ = 0 and *α_zx_* = 0, respectively, which indicates that the bubble oscillates in situ in this time period; meanwhile, the bending angle *βzx* < 0° of the C-1 plate indicates that the free end of the C-1 plate is in the process of subsidence, and the value decreases first and then increases. When the time is *t* = 4.83–8.00 ms, values of *r*_1_ = 0 and *α_zx_* < 90° indicate that the bubble moves downward to the left; the bending angle (*β_zx_*) of the C-1 plate is less than 0° at first, and then greater than 0° after *t* = 6.0 ms, indicating that the free end of C-1 plate changes from a sinking state to an upward state. The trajectory of the bubble and the deformation of the C-1 plate are observed quantitatively in the lateral view. It was found that the trajectory of the bubble is the same as that of the C-1 plate in a twisting state; that is, the bubble moves vertically downward, and the plate has no twisting deformation. It was also found that the length and deflection angle (*r*_1_, *α_zx_*) of the bubble trajectory are divided into two branches when the time is 4.5 ms: one group has values of *r*_1_ > 0 and 90° < *α_zx_* < 180°; and the other group has values of *r*_1_ > 0 and *α_zx_* < 90°, which indicates that the bubble is divided into two parts. The former moves to the upper left, and the latter to the lower left.

For the side view projection of *γ* = 1.00, the value of *β_zx_* = 0° for the C-1 plate indicates no twisting deformation; the length and deflection angle (*r*_2_, *α_yz_*) of the bubble track are also divided into upper and lower parts. Through the above analysis, it can be seen that under the bending effect of the C-1 plate, the bubble does not move in the first period, and after collapse, its trajectory is no longer vertical up and down in the projection of the front view but instead it makes a certain angle with the direction of gravity; however, the trajectory of the bubble still moves vertically up and down under the projection of the side view.

**Figure 12:**
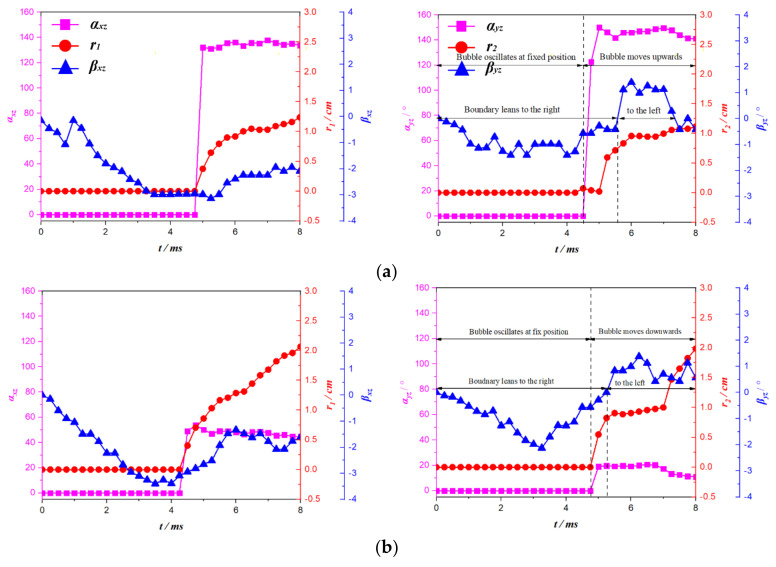

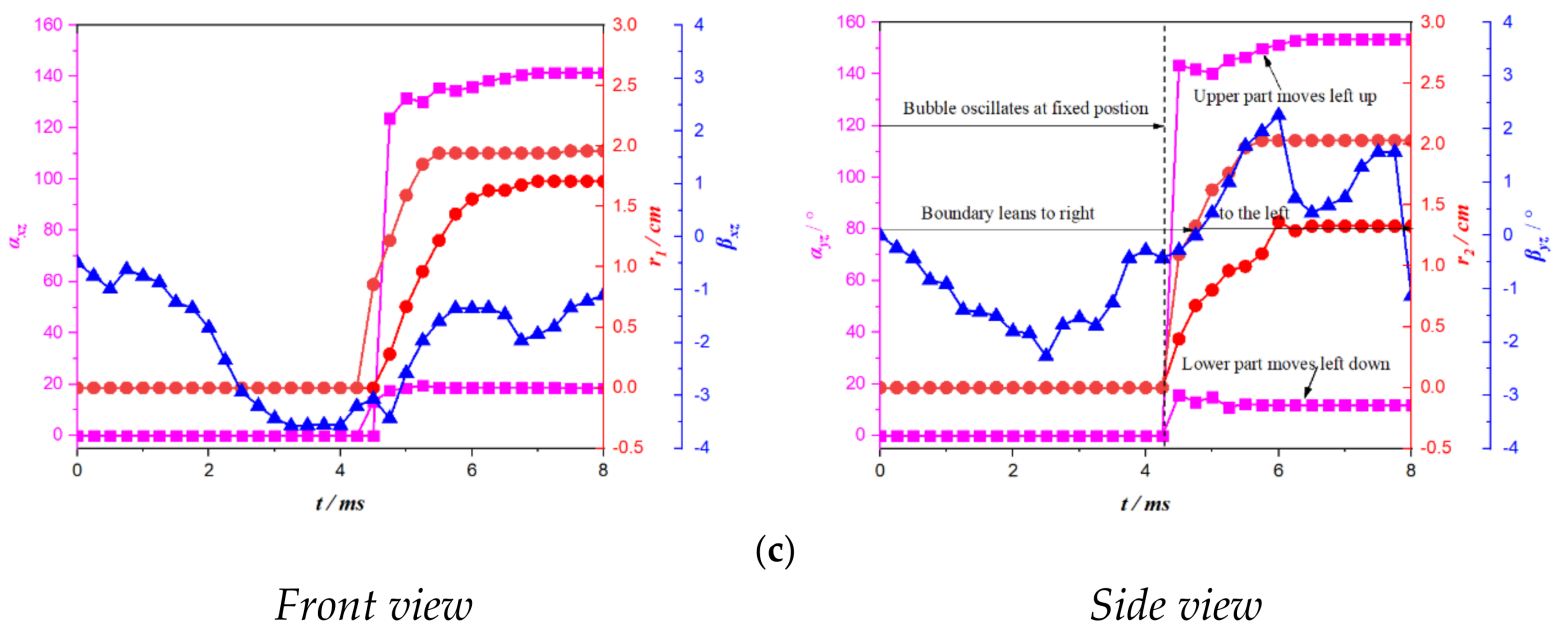
Effect of bending-twisting coupling effect of C-2 plate at different initial positions on bubble migration direction. (**a**) *γ* = 1.65, (**b**) *γ* = 1.32, (**c**) *γ* = 1.00.

Figure 12 shows the effect of the bending-twisting coupling effect on the bubble migration direction of the C-2 plate at different initial positions of the bubble. When the initial position of the bubble is *γ* = 1.65, 1.32, and 1.00, it was found that the trajectory of the bubble and the deformation of the C-2 plate in the front view are very similar to those of the C-1 plate. The reason for this is that the migration path of the bubble on the front view projection is curved due to the bending deformation of the plate. Therefore, the front view of the C-2 plate will not be analyzed in detail. When the initial position of the bubble is *γ* = 1.65, the trajectory of the bubble and the deformation of the C-2 plate are projected on the side view. The values of *r*_2_ = 0 and *α_yz_* = 0° indicate that there is no movement of bubble in this period; the twisting angle of *β_zx_* < 0° for the C-2 plate is less than 0°, which indicates that the C-2 plate has obvious twisting deformation, and the left endpoint of the plate is higher than the right endpoint. When the time is *t* = 4.50–8.00 ms, the length and deflection angle of the bubble trajectory are *r*_2_ > 0 and 90° < *α_yz_* < 180°, indicating that the bubble moves upward to the left; the twisting angle of the plate is *β_zx_* > 0° after *t* = 5.8 ms, indicating that the left endpoint of the bubble is lower than the right endpoint.

For the condition *γ* = 1.32, the twisting trend of the C-2 plate in the whole process is similar to that of the condition *γ* = 1.65: when the time is *t* = 0–4.8 ms, the bubble center remains unchanged; when the time is *t* = 4.8–8.0 ms, the length and deflection angle of the bubble trajectory are *r*_2_ > 0 and *α_yz_* < 90°, indicating that the bubble moves downward to the left.

For the condition of *γ* = 1.00, the motion of the bubble is more complex. In the first period (*t* = 0–4.5 ms), the bubble center remains unchanged; however, when *t* = 4.5–8.0 ms, the length and deflection angle (*r*_1_, *α_xz_*) of the bubble trajectory are divided into two branches, one group with values of *r*_1_ > 0 and 90° < *α_xz_* < 180° and the other group with values of *r*_1_ > 0 and *α_xz_* < 90°, indicating that the bubble is divided into two parts. The former moves to the upper left, and the latter to the lower left. At the same time, the twisting angle of the plate is the same as that of the working conditions for *γ* = 1.65 and 1.32.

**Figure 13:**
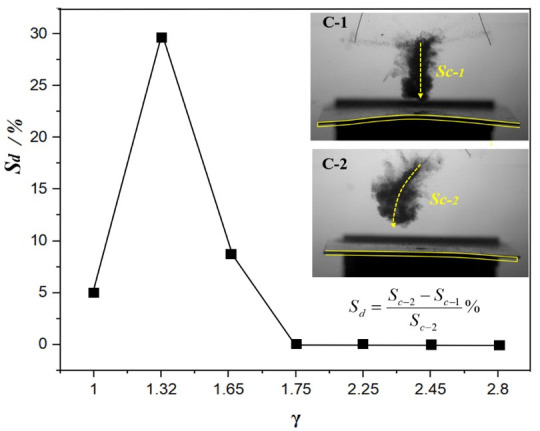
Bubble migration distance ratio near C-1 and C-2 plates.

In order to further study the difference between the bending-twisting coupling effect of the C-2 plate and the bending effect of the C-1 plate on the path of bubble migration, Figure 13 shows the curve of the bubble migration path ratio (*S_d_*) near the C-1 and C-2 plate with the different initial positions of the bubble. The data show that the distance of bubble migration near the C-2 plate is more than that near the C-1 plate; this data was obtained by calculating the mapping trajectory of the bubble migration curve in the side view. As shown in the figure, when the initial distance of the bubble is *γ* = 1.00, the distance ratio is *S_d_* = 5%; when the initial distance is increased to *γ* = 1.32, the distance ratio increases to *S_d_* = 30%; and when *γ* = 1.65, the distance ratio decreases to *S_d_* = 8%. After that, the distance ratio is almost zero. This is because when the bubble is close to the plate, the bubble adheres to the surface of the wall structure, and the bubble cannot move. However, when the initial distance is far away, the force exerted by the plate on the bubble is very small, and the bubble almost collapses in Rayleigh oscillation. It can be seen from the figure that when the bubble is between 1.00 < *γ* < 1.75, the migration of the bubble will present a curve track due to the bending-twisting coupling effect of the anisotropic composite plate, which increases the distance for the bubble to reach the structural wall and significantly consumes the kinetic energy of the high-speed fluid generated by the collapse of the bubble, thus reducing the impact of the high-speed fluid on the structural wall.

### 3.4. Mechanism Analysis

**Figure 14:**
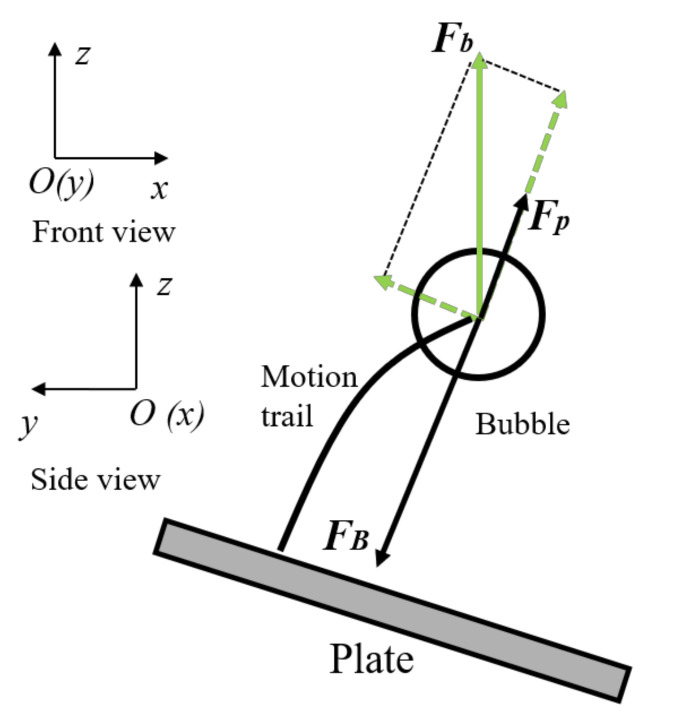
Mechanism analysis of bubble curve migration near bending-twisting coupled plate.

In order to explain the curvilinear migration of a bubble near an anisotropic composite plate under bending-twisting coupling, Figure 14 shows the Bjerknes force *(F_B_*), buoyancy (*F_b_*), and oscillating force (*F_P_*) near the deformed plate. This figure is applicable to both the front view and the side view. When the bending-twisting coupling effect occurs in anisotropic composite plate, the coupling effect of bending and twisting deformation will occur. Because the principle of force analysis is similar, we only took the side view (*y-o-z*) as an example. According to the mirror image principle, when the bubble oscillates near the wall, it is subjected to the combined action of the Bjerknes force, buoyancy force, and oscillating force of the plate. The direction of buoyancy is vertically upward; the Bjerknes force and the oscillating force of the plate follow a straight line perpendicular to the wall structure, where the Bjerknes force points to the wall and the direction of the oscillating force changes with the deformation of the plate. Because the order of magnitude of the buoyancy force and oscillating force is much smaller than the Bjerknes force, the Bjerknes force plays a dominant role. In addition, as the twisting angle of the plate changes with time, the angle of the Bjerknes force also begins to change; thus, the bubble follows a curve migration movement.

## 4. Conclusions

In these experiments, the effects of anisotropic laminated composite plates on the dynamics of bubbles were investigated using a high-speed camera system. The two laminated composite plates, fixed as a cantilever beam, had different ply angles, namely 0° and 30°. The main findings are as follows:

The bubble migration near the 30° plate had an evident titled angle during the collapse and rebound stages, which is different from the 0° plate where all bubbles moved vertically upward.

A characterization method of bubble migration was proposed to quantitatively describe the curved bubble trails and the deformation of composite beams in temporal and spatial scales.

The bubble migration path was determined by the dynamic response of composite beams. The laminated composite beam with 30° presented a significantly greater bending-twisting coupling phenomenon subjected to the action of bubble collapse as compared with the 0° plate.

Regarding future works, a numerical fluid–structure interaction method should be proposed to calculate the detailed information about fluid structure (i.e., pressure and velocity) and deformation of composite beams (i.e., strain and stress distribution), in order to deeply analyze the mechanism of the interaction between bubble dynamics and anisotropic beams.

## Figures and Tables

**Table 1:** Mechanical properties of carbon fiber plate.

Along the Fiber Orientation		Perpendicular to Fiber Orientation		Shear Modulus
*E*_1_/GPa	*v*_12_	*E*_2_/GPa	*v*_21_	*G*_12_/GPa
42.83	0.305	6.48	0.045	5.48

## Data Availability

Not applicable.
